# Determination of the mimic epitope of the M-like protein adhesin in swine *Streptococcus equi *subsp. *zooepidemicus*

**DOI:** 10.1186/1471-2180-8-170

**Published:** 2008-10-07

**Authors:** Hongjie Fan, Yongshan Wang, Fuyu Tang, Chengping Lu

**Affiliations:** 1 College of Veterinary Medcine, Nanjing Agricultural University, Nanjing 210095, PR China; 2 Institute of Veterinary Medicine, Jiangsu Academy of Agricultural Sciences, Nanjing 210014, PR China

## Abstract

**Background:**

The M-like protein, also known as SzP, is expressed on the surface of *Streptococcus equi *subsp. z*ooepidemicus *(*S*. z*ooepidemicus*). Previous studies demonstrated that SzP is similar to M protein of group A *Streptococcus *in the structure and characteristics of antiphagocytosis. The M protein is an adhesin that can bind to the host cells, however it is not known whether the SzP of *S*. z*ooepidemicus *also functions as an adhesin. We conducted an investigation to determine SzP as an adhesin, and one SzP epitope was identified to be responsible for mediating binding to HEp-2 cells.

**Methods:**

The gene encoding SzP was expressed in *E. coli*, and the purified recombinant SzP (rSzP) was recognized by rabbit anti-*S*. z*ooepidemicus *antibodies using immunoblot. Furthermore, the adherence of *S*. z*ooepidemicus *to HEp-2 cells was inhibited by anti-rSzP antibodies in a dose-dependent manner. We employed a random 12-peptide phage display library for screening of immunodominant mimics of the SzP, which were recognized by an anti-SzP specific monoclonal antibody (mAb 2C8). Initial positive phage clones were identified by ELISA, followed by assays to determine the adherence-inhibiting ability of the peptide.

**Results:**

Ten out of fourteen selected positive clones showed high reactivity that effectively inhibited the binding of mAb 2C8 to rSzP. The motif XSLSRX was highly conserved among six of the ten clones.

**Conclusion:**

Collectively, our findings suggest that the motif XSLSRX may represent an immunodominant mimic epitope of the SzP of *S*. z*ooepidemicus *strain ATCC 35246, and that the same epitope may be used to mediate SzP binding to HEp-2 cells.

## Background

*Streptococcus equi *subsp. z*ooepidemicus *(*S*. z*ooepidemicus*), which belongs to Lancefield group C streptococci, is an important animal pathogen, especially in horse [1]. It has a broad host spectrum and occasionally infects humans. Human infections may occur following ingestion of unpasteurized milk or dairy products [2], or after contact with pigs [3]. In China, *S*. z*ooepidemicus *is the main pig pathogen. In the summer of 1975, a *S*. *zooepidemicus *disease outbreak occurred among pigs in the Sichuan province, China. Clinical symptoms of the diseased pigs included painful swelling of the joints, respiratory disturbances, and diarrhea. More than 300,000 pigs died within two weeks. According to bacteriological examinations, *S*. z*ooepidemicus *was isolated from most of the diseased pigs, and it appeared to be one of the major causative agents [4].

M protein is an important virulence factor of group A streptococci: this fibrillar, surface-exposed protein deters opsonization of the organism using the alternate complement pathway [5,6]. Strains of group A streptococci expressing M protein are resistant to phagocytosis by human polymorphonuclear leukocytes, whereas strains that fail to express M protein are avirulent [7]. Previous studies demonstrated that *S*. z*ooepidemicus *carry antigens with characteristics of the antiphagocytic M protein of the Lancefield group A and G streptococci; it was named M-like protein (SzP) [8]. Cloning of the gene encoding SzP from an equine strain of subsp. *zooepidemicus *revealed a single open reading frame of 1,128 bp [9], whereas the SzP gene (GenBank accession number: AY263781) from a pig strain of subsp. *zooepidemicus *showed a single open reading frame of 1,137 bp (GenBank accession number: U04620) [10].

Similar to other streptococcal species, *S*. *zooepidemicus *binds to a number of host proteins, including immunoglobulin G [11], serum albumin [12], fibronectin, collagen [13], and α-macro-globulin [14] through cell surface components. These host-parasite recognition components may function as virulence factors by acting as adhesions or antiopsonins. Bacterial adhesion to the host cell is the first step in bacterial infection. The M protein of streptococcus group A is an adhesin that can bind to the host cell, however it is not known whether the SzP of *S*. z*ooepidemicus *also functions as an adhesion, and the adhesion mechanism of SzP binding to host cell is unclear. Here, we report the adhesive function of the SzP to human HEp-2 epithelial cells. Furthermore, we identified several immunodominant mimic epitopes of SzP using random peptide phage library in combination with ELISA and binding-inhibition assays. Alignment of the phage display peptides yielded a conserved motif, XSLSRX, which represents an immunodominant mimic epitope of the SzP of *S*. z*ooepidemicus *strain ATCC 35246 and may mediate binding to HEp-2 cells.

## Results

### Molecular characterization of M-like protein

**Figure 1:**
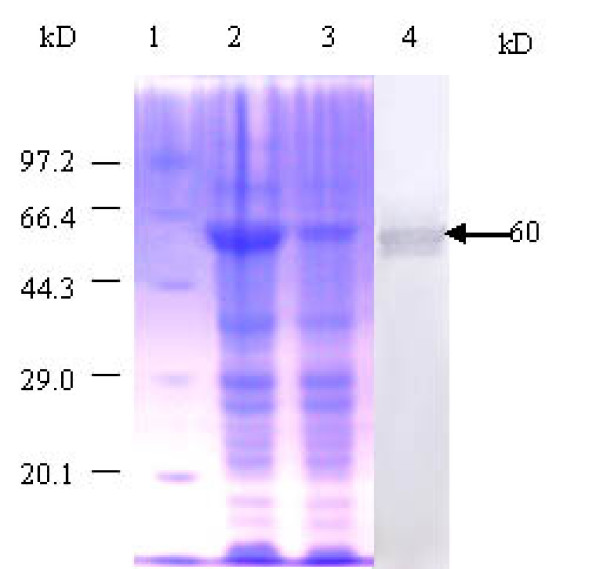
**SDS-PAGE analysis and western blot of rSzP**. Lane 1, Marker. Lane 2, *E. coli *BL21 (DE3) pLysS after IPTG induction. Lane 3, *E. coli *BL21 (DE3) pLysS before IPTG induction. Lane 4, Reactivity of purified rSzP with rabbit anti-*S. zooepidemicus *antibody.

Nucleotide sequence analysis revealed a single open reading frame in the *szp *gene of *S. zooepidemicus *ATCC 35246 isolated from pigs; translation of this open reading frame revealed a protein of 379 amino acids. The *szp *gene showed 86.9% homology at the nucleotide level with the *szp *gene of *S. zooepidemicus *W60 strain isolated from the horse, and 29.4% homology with the M protein gene of group A streptococci [10]. Expression of the mature *szp *gene under the control of the T7 promoter sequence was achieved with high yield and purity (Fig. 1). SDS-PAGE analysis demonstrated that the recombinant protein was 60 kDa, and was confirmed to be SzP by immunoblot analysis using rabbit anti- *S. zooepidemicus *antibody.

### Binding of rSzP to HEp-2 host cells

**Figure 2:**
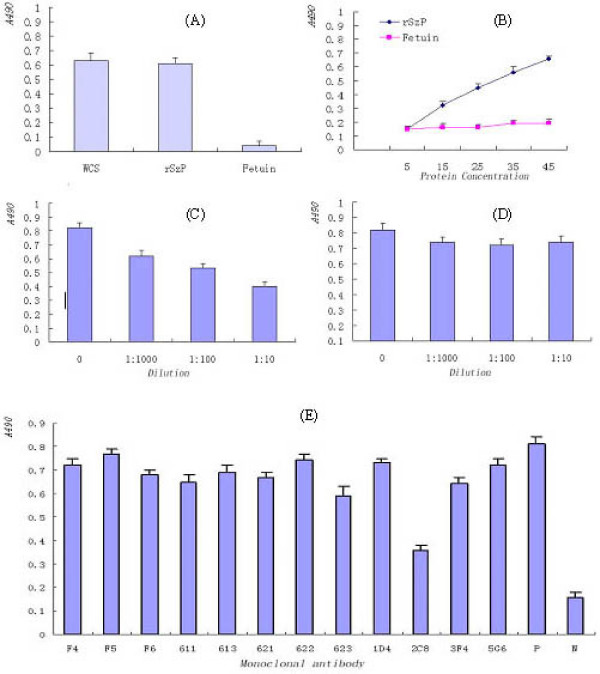
**Characterization of purified rSzP protein binding to HEp-2 cells by ELISA**. (A) Binding of biotinylated S. zooepidemicus whole cells, rSzP protein, and a fetuin control to HEp-2 monolayers. (B) Dose-dependent binding of rSzP to HEp-2 cells. (C) Inhibition of *S. zooepidemicus *binding to HEp-2 monolayers following pretreatment of bacteria with rabbit polyclonal anti-SzP antibodies. (D) No inhibition of HEp-2 binding with control rabbit serum. (E) Inhibition of *S. zooepidemicus *binding to HEp-2 monolayers following pretreatment of bacteria with monoclonal antibodies against SzP. For all experiments, absorbance values are means from representative experiments performed in triplicate. Error bars show the range of absorbance values.

The binding of purified rSzP to HEp-2 monolayers was assessed by ELISA. Purified rSzP exhibited significant, dose-dependent binding to HEp-2 cells, whereas binding of the negative control, fetuin, was negligible (Fig. 2A and 2B). Adherence inhibition assays were used to determine the ability of anti-SzP antibodies to inhibit adherence of *S. zooepidemicus *to HEp-2 cells. Biotinylated *S. zooepidemicus *intact cells were first pretreated with the anti- SzP polyclonal/monoclonal antibodies and were then tested for the ability to bind HEp-2 monolayers. Pretreatment of biotinylated *S. zooepidemicus *with increasing amounts of anti- SzP polyclonal antibodies resulted in a significant (P < 0.01) dose-dependent inhibition of bacterial adherence (23%, 48%, and 58% reduction at 1:1,000, 1:100, and 1:10 dilutions, respectively) (Fig. 2C). No significant difference (P > 0.05) was observed in the adherence of normal serum-treated bacteria to HEp-2 cells (Fig. 2D). Out of the 12 monoclonal antibodies,only 2C8 was able to inhibit the adherence of *S. zooepidemicus *to HEp-2 cells (Fig. 2E).

### Biopanning

The anti-SzP monoclonal antibody 2C8 was immobilized onto microtiter wells to pan mimic epitopes from random 12-mer peptides library during five successive rounds of selection. The productivities of the target phages for the first and the fifth biopanning were 1.71 × 10^-6 ^and 3.39 × 10^-6^, respectively, and false positive rates were reduced from 0.203 to 2.2 × 10^-3^, respectively. Taken together, these values indicate a significant enrichment of the selected phages.

### Specificity analysis

**Figure 3:**
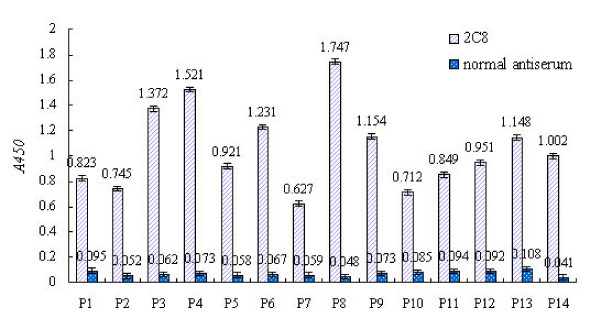
**ELISA reactivities of 14 phage clones (P1–P14) with mAb 2C8 and normal antiserum**. ELISA experiments are described in the Materials and Methods. In all experiments, absorbance values are means from representative experiments performed in triplicate. Error bars show the range of absorbance values.

ELISA was used to confirm that the phage-displayed peptides were specifically recognized by mAb 2C8; normal antiserum was used as a negative control. A phage was considered to be specifically bound by mAb 2C8 when (*Aa*:*Ab) *> 0.3, where *Aa *and *Ab *correspond to the absorbance of the phage binding to 2C8 mAb and normal antiserum, respectively. Based on these criteria, 14 phage colonies were positive in a sandwich ELISA assay (Fig. 3) and were propagated for inhibition experiments.

### Inhibition experiments

**Figure 4:**
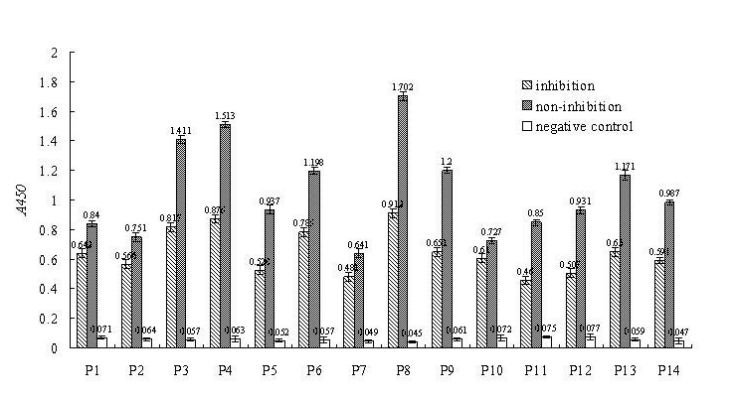
**Inhibition assays of 14 phage clones (P1–P14) with rSzP**. Inhibition of phage binding to mAb was detected by ELISA as described in Section 2. For each phage, binding of phage to mAb was inhibited by rSzP. Average values (A450 nm) from three independent experiments have been determined. Results are expressed as the difference between the average value of non-inhibition and that of inhibition.

Competitive inhibition experiments were performed to determine whether the selected phages contained the same or mimic epitopes of the SzP. The results showed that ten of the fourteen clones (P3, P4, P5, P6, P8, P9, P11, P12, P13, and P14) displayed an inhibition level of greater than 40% by mAb 2C8 (Fig. 4). These results demonstrated that each selected phage contained a binding site to the mAb, that the binding sites of the phage-displayed peptides are SzP-specific, and that they may function as immunodominant mimics of the SzP (Fig. 4).

### Characterization of positive selected phage clones

Ten phages that showed specific binding were selected from an initial 14 phages and sent for sequencing. Sequencing of the phage inserts revealed that four out of the 10 phages displayed KSLSRHDHIHHH, one phage displayed SSLSRGKPSRP, one phage displayed SSLSPQRHSYPA, and only one phage (P11) showed no similarity to either peptide sequence (Table 1). Alignment using the DNAStar-MegAlign software showed that the motif XSLSRX was conserved among them.

**Table 1:** Inserts of phage clones from the fifth round of panning with mAb 2C8.

Clone number	Peptide sequence	Frequency
P3, P4, P8, P13	**KSLSR**HDHIHHH	4
P14	**SSLSR**GKPSPRP	1
P6	**PSLSR**Q**R**HS**Y**PA	1
P5	GTV**L**PD**LSR**NPY	1
P9	TW**L**TP**S**FPFYLG	1
P12	TP**L**D**R**GTWLFTK	1
P11	NTFNEFWPSNSW	1

## Discussion

M protein is composed of two predominantly alpha-helical protein chains assembled in a coiled-coil that extends from the bacterial cell surface [15]. The M protein C terminus, which is anchored to the cell membrane and traverses the cell wall, is highly conserved, whereas the N terminus, which contacts the external environment, is highly variable and accounts for the type specificity. There are more than 100 known M protein serotypes of group A streptococci, and the serotype specificity is largely determined by epitopes located in the N-terminal 40 to 50 residues [16-19]. SzP is similar in structure to M protein of group A streptococci. In addition to *S*. z*ooepidemicus*, SzP have also been detected in other group C and G streptococci [10,20,21]. SzP elicited protective antibodies and impaired deposition of C3 on the bacterial surface [9]. *szp *gene has also been cloned from *S*. z*ooepidemicus *[9,10]. The predicted amino acid structure contains an alpha-helical repeat and shows highly homology to the C terminus of M proteins of group A, however the extensive A, B, C repeat regions found in M proteins of group A are absent. When compared to the SzP of *S*. z*ooepidemicus *equine strain W60, the following N-terminal residues differ: S27G, S28V, V30A, and A32S; all of the swine strains shared the same signal peptide. The C terminus of SzP of *S. zooepidemicus *is rich in proline residues, and is composed primarily of PEPK repeats. The known swine strains shared 11 PEPK repeats with the SzP, whereas the equine strain W60 shared only 10 PEPK repeats [9,22].

Bacterial attachment to a host tissue is often the first step leading to colonization and the development of an infectious disease. The bacteria are dependent on their own surface components, called "adhesins", for binding in a stereochemical manner to complementary molecules on the host tissue surface. Most bacterial adhesins are proteins, and they are frequently contained in filamentous surface appendages known as fimbriae or pili [23,24]. Streptococcal survival in the host depends on the M protein, which protects the bacterium from phagocytosis by polymorphonuclear leukocytes [7]. Bacterial strains that express high levels of M protein have been reported to adhere to human epithelial cells in significantly higher numbers than M protein-deficient strains, suggesting that this molecule also plays a critical role in adherence to human tissue [25]. The present study has shown that rSzP can attach to HEp-2 cells in a manner inhibited by both anti-rSzP antibodies and by anti-*S*. z*ooepidemicus *antibodies, suggesting that the SzP is a putative adhesin of *S*. z*ooepidemicus*, which may contribute to the colonization *in vivo*.

Recent investigations have successfully employed phage display libraries to identify specific ligands for antibodies, enzymes, cell surface receptors, drugs, and epitopes [26-28]. Furthermore, studies have demonstrated that some selected ligands do not necessarily resemble the natural ligand, but instead mimic their binding properties [29]. In view of this, peptide phage libraries may be used as a rapid and convenient method for selecting "novel" peptide epitopes regardless of whether their displayed peptide sequences are homologous to the natural antigen, and may thereby serve as ideal candidates in vaccine development [27]. The potential for use of random peptide phage display libraries in defining specificities of mAbs has been demonstrated successfully [28]. In the present study, following five rounds of enrichment, 14 positive phage clones had higher binding capacity, indicating that the ligands had been selected successfully. Inhibition assays were used to assess whether ligands obtained in this study mimicked the SzP. Results showed that 10 phage clones effectively inhibited mAb 2C8 binding to rSzP. The motif XSLSRX was identified as highly conserved among these peptides. We believe that these findings will be helpful in clarifying the pathogenic mechanism of *S. zooepidemicus*. However, we did not find similar motifs in the SzP. Thus, the XSLSRX motif might act as a mimic epitope. Future studies are needed to address whether the XSLSRX epitope may be discontinuous.

## Conclusion

The investigation provided strong evidence that the M-like protein of *Streptococcus equi *subsp. *zooepidemicus *strain ATCC 35246 was an adhesin. The motif XSLSRX may represent an immunodominant mimic epitope of M-like protein, and that the same epitope may be used to mediate M-like protein binding to HEp-2 cells.

## Methods

### Bacterial strains, plasmids, and growth conditions

*S. zooepidemicus *strain ATCC 35246 was isolated from a diseased pig in Sichuan province in 1975 and was stored at -70°C in Todd-Hewitt broth (THB). The strain was grown on Todd-Hewitt agar plates supplemented with 5% sheep blood at 37°C. *Escherichia coli *strain BL21 (DE3) pLysS was grown in Luria-Bertani broth. The PET32a(+) vector was used for subcloning and protein expression.

### S. zooepidemicus M-like protein expression and purification

The mature *szp *gene of ATCC 35246 (without the putative promoter or leader signal peptide) was directionally subcloned downstream of an isopropyl β-D-1-thiogalactopyranoside (IPTG)-inducible T7/*lac *promoter, and the start codon (ATG) of the PET32a(+) expression vector was used to produce recombinant SzP (rSzP). The resulting plasmid was then used to transform competent *E. coli *BL21 (DE3) pLysS as described previously [15]. Expression was induced by the addition of IPTG, and the fusion protein was purified using a nickel-loaded affinity chelate resin purification system (Tosoh Biosep, Montgomeryville, PA, USA) according to the manufacturer's instructions.

Expression of rSzP was determined by sodium dodecyl sulfate polyacrylamide gel electrophoresis (SDS-PAGE) analysis using whole-cell lysates before and after IPTG induction. Immunoblot analysis was performed as described previously [16] to determine whether the rSzP protein was recognized by antiserum against *S. zooepidemicus*.

### Preparation and characterization of monoclonal antibodies

Briefly, BALB/c mice were intraperitoneally immunized twice (on days 1 and 14) with 20 μg of purified rM-like protein (10 μg each), first in complete adjuvant and then in incomplete Freund's adjuvant. Two weeks after the second immunization, the mice were boosted intraperitoneally with 5 μg of purified rSzP protein in phosphate-buffered saline (PBS). Spleens from the mice were collected 3 days after the boost and were used for fusion with SP2/0 myeloma cells following a standard polyethylene-glycol-mediated cell fusion procedure. Hybridomas secreting antibodies were screened by an in-house *S. zooepidemicus*-specific indirect ELISA, and the positives were cloned by limiting dilution,ELISA tests were performed as described previously [17]. Twelve SzP-specific mAbs, including F4, F5, F6, 611, 613, 621, 622, 623, 1D4, 2C8, 3F4, and 5E6 were obtained. Their immunoglobulin subclasses were determined using a subtyping kit (Pierce Biotechnology, Rockford, USA), and were IgG1 for F4, 611, 613, 623, 2C8, and 3F4, IgG2b for F6, 621, 622, and 5E6, and IgM for F5 and 1D4. Competitive ELISA revealed that the twelve mAbs recognized spatially independent epitopes. The mAbs in ascites fluids were purified by saturated ammonia sulfate precipitation and DEAE-cellulose chromatography as described previously [17].

### Preparation of polyclonal antibodies

Polyclonal antibodies (pAb) against SzP were prepared using 2-month-old New Zealand rabbits. Rabbits were first injected subcutaneously in three sites with a total of 2 ml emulsified mixture (PBS containing 200 μg of purified protein) and complete Freund's adjuvant (Sigma, St. Louis, MO, USA). Booster inoculations were given at biweekly intervals in an identical manner, except incomplete Freund's adjuvant (Sigma, St. Louis, MO, USA) was used. Blood was drawn at each boost for antibody monitoring. When the serum anti-SzP titer reached 1:10,000 as measured by indirect ELISA, rabbits were sacrificed by cardiac exsanguinations, and antiserum was prepared. IgG from the rabbit antiserum was purified by saturated ammonia sulfate precipitation and DEAE-cellulose chromatography. At the same time, anti-serum against *S. zooepidemicus *was prepared as described above.

### Binding of purified rSzP to HEp-2 cells

Binding tests were performed as described [18]. Briefly, HEp-2 cells were seeded (1.4 × 10^4 ^cells/well) in growth medium containing 10% fetal bovine serum (ATCC) in microtiter wells precoated with 0.2% gelatin and were grown to confluence in a humidified atmosphere at 37°C with 5% CO_2_. The following day, the seeded wells were washed three times with fetal calf serum-free M1640 medium, and either live *S. zooepidemicus *(1.4 × 10^5 ^cells/well), purified rSzP, or a fetuin control (3 μg/well) diluted in M1640 medium was added to the wells and incubated for 4 h in a humidified atmosphere at 37°C with 5% CO_2_. The plates were washed three times as indicated above and were fixed in 20% acetone (150 μl) for 10 min at room temperature. Excess acetone was removed from the wells, and the plates were allowed to dry at 37°C overnight. For immunoassays, the acetone-fixed cells were probed with rabbit anti-*S. zooepidemicus*, rabbit anti- rSzP, or rabbit anti-fetuin (Sigma, St. Louis, MO, USA; 1:1,600) diluted in PBS containing 0.5% Tween-20 (PBS-T), followed by incubation with horseradish peroxidase (HRP)-conjugated goat anti-rabbit IgG (Sigma, St. Louis, MO, USA; 1:8,000 diluted in PBS-T). Negative controls were used in all experiments and included wells containing only cells and wells containing all components except primary antibody, secondary antibody, or fetuin. Experiments were performed twice and in triplicate. Treatment differences were tested by student's t-test [19].

For adherence inhibition, biotinylated bacteria (1.4 × 10^5 ^cells/well) were first pretreated with specific anti-rSzP antibodies (1:100 diluted in PBS-T) for 1 h at 37°C and were washed before being used to infect the monolayers as described above. Bacteria were biotinylated using the EZ-Link Sulfo-NHS-LC-Biotinylation kit (Pierce, Rockford, IL, USA) according to the manufacturer's instructions. Bacteria preincubated with normal rabbit serum were used as controls, and wells receiving no bacteria were used as blanks. Additional controls included wells containing all ingredients except primary or secondary antibodies. Binding of biotinylated bacteria to the monolayers was detected using HRP-conjugated streptavidin (Pierce, Rockford, IL, USA) and a substrate chromogen mixture consisting of 0.01% hydrogen peroxide in 0.1 μg/ml tetramethyl benzidine (TMB) (Sigma, St. Louis, MO, USA). The ability of the anti-SzP mAbs to inhibit adherence was determined as described above.

### Affinity selection and panning

We used the Ph.D.-12™ phage display peptide library kit (New England Biolabs, Bevery, MA, USA), which displays peptide 12-mers at the N-terminus of pIII of M13 phage with a complexity 2.7 × 10^9 ^transformants. A biopanning protocol was employed according to the manufacturer's instruction. Briefly, 96-well microtiter plates (Nunc, Roskilde, Denmark) were coated with the target mAb 2C8 overnight at 4°C and were incubated with blocking solution for 1 h at 37°C. A solution containing the phage-display peptide library (1.5 × 10^11 ^virions) was added to the coated wells and incubated for 1 h at 37°C. Unbound phages were removed by 10 washes with Tris-buffered saline containing Tween-20 (TBST), bound phages were eluted in 100 μl elution buffer [0.2 M glycine-HCl (pH 2.2), 1 mg/ml BSA], and the eluate was neutralized by the addition of 15 μl 1 M Tris-HCl (pH 9.1). The phage outcome was calculated by infecting ER2738 bacteria followed by counting of transducing units (TU) on LB/IPTG/X-gal plates. Subsequently, the phage eluates were amplified and concentrated using PEG/NaCl precipitation. Ten microliters of amplified elute was prepared for the next round of panning. The panning procedure of the following four generations was similar to that of the first panning, except the Tween-20 concentration in the TBST was increased to 0.5% (V/V) in the washing step. The number of phages added was designated as input, the eluted phages were designated as output, and the unbound phages that were removed during the 10 wash steps were designated as washing.

### Specificity analysis

Sandwich ELISA was performed as follows. Wells in 96-well microtiter plates were coated with the anti-SzP mAbs solutions (100 μl each), which were purified from ascitic fluids and diluted to 5 μg/ml in coating buffer (0.05 M sodium carbonate, pH 9.7), and incubated overnight at 4°C. The wells were blocked by incubation with 200 μl 1 % BSA in PBS-T for 1 h at 37°C. After the wells were washed three times with PBS-T, 100 μl samples of each amplified single colony phage culture (resuspended in PBS) were added to each well, followed by incubation for l h at 37°C. After three washes with PBS-T, 100 μl HRP-conjugated anti-M13 monoclonal antibody (Amersham Pharmacia Biotech Inc, Piscatway, NJ, USA) diluted 1:2,000 in PBS-T containing 1% BSA was added to each well, followed by incubation for 1 h at 37°C. After three additional PBS-T washes, 100 μl of the substrate TMB was added to each well, followed by a 10 min incubation. Finally, 100 μl of 2 M sulfuric acid was added to each well to stop the reaction. Relative to wells containing phages without peptide inserts, absorbance values equal to or greater than two-fold higher were considered positive. All phage clones were tested in triplicate.

Competitive inhibition ELISA was performed to test the specificity of the peptides displayed on the selected phages. Briefly, 96-well microtiter plate wells were coated with anti-SzP mAb (2C8) solution (1:100 diluted in PBS-T) incubated overnight at 4°C and blocked. At the same time, 1 × 10^12 ^virions for each phage clone and 100 μl purified rSzP (20 μg/ml in PBS) were mixed separately and incubated for 30 min at 37°C. The mixtures were then added to the wells coated with anti-SzP mAbs, followed by incubation for l h at 37°C; positive control wells were incubated with phages, and negative control wells were incubated with PBS. After three washes with PBS-T, 100 μl HRP-conjugated anti-M13 monoclonal antibody (Amersham Pharmacia Biotech Inc, Piscatway, NJ, USA) diluted 1:2,000 in PBS-T containing 1% BSA was added to each well, followed by 1 h incubation at 37°C. The subsequent steps were performed as described above for the sandwich ELISA. If the selected phage contained mAb-specific epitope/mimotopes, the binding of the coated mAb with the selected phages would be competitively inhibited by the rSzP in solution. All phage clones were tested in triplicate.

### Determination of peptide sequences

Phage-containing supernatants from a single positive clone were collected for purification of sequencing templates. PEG/NaCl (200 μl) was added to 500 μl of phage-containing supernatant, followed by mixing and incubation at room temperature for 10 min. Following centrifugation, the pellet was resuspended in 100 μl iodide buffer [100 mM Tris-HCl (pH 8.0), 1 mM EDTA, 4 M NaI], 250 μl ethanol was added, and the resuspended pellet was incubated for 10 min at room temperature followed by centrifugation. The pellet was washed with 70% ethanol, dried, and dissolved in TE buffer [10 mM Tris-HCl (pH 8.0), 1 mM EDTA]. The sequencing primer used was 5'-CCCTCATAGTTAGCGTAACG-3'. DNA sequences were translated into peptide sequences according to the manufacturer's instructions (Ph.D.-12™, New England Biolabs, Bevery, MA, USA).

## Authors' contributions

HjF participated in the preparation of monoclonal antibodies and polyclonal antibodies, Carried out the binding tests and drafted the manuscript. YsW contributed to the determination of immunodominant mimic epitope of M-like protein.  FyT carried out the expression of M-like protein. CpL supervised the design of the study and contribution to the writing of the article.
